# BOTNet: Deep Learning-Based Bearings-Only Tracking Using Multiple Passive Sensors

**DOI:** 10.3390/s21134457

**Published:** 2021-06-29

**Authors:** Hadar Shalev, Itzik Klein

**Affiliations:** Department of Marine Technologies, University of Haifa, Haifa 3498838, Israel; kitzik@univ.haifa.ac.il

**Keywords:** bearings-only, target tracking, autonomous underwater vehicle, deep learning

## Abstract

Bearings-only target tracking is commonly used in many fields, like air or sea traffic monitoring, tracking a member in a formation, and military applications. When tracking with synchronous passive multisensor systems, each sensor provides a line-of-sight measurement. They are plugged into an iterative least squares algorithm to estimate the unknown target position vector. Instead of using iterative least squares, this paper presents a deep-learning based framework for the bearing-only target tracking process, applicable for any bearings-only target tracking task. As a data-driven method, the proposed deep-learning framework offers several advantages over the traditional iterative least squares. To demonstrate the proposed approach, a scenario of tracking an autonomous underwater vehicle approaching an underwater docking station is considered. There, several passive sensors are mounted near a docking station to enable accurate localization of an approaching autonomous underwater vehicle. Simulation results show the proposed framework obtains better accuracy compared to the iterative least squares algorithm.

## 1. Introduction

Bearings-only target tracking is commonly used in several domains, such as air [1,2], space [3], and underwater [4]. In this method, passive sensors provide line-of-sight (LOS) measurements from the sensor to the target. The LOS is a function of sensor-target azimuth and elevation angles. Those angles are nonlinear functions of the target position in Cartesian coordinates and the sensor positon. Thus, with a single LOS measurement, localization of a target cannot be achieved since each LOS has two measured angles and the number of unknown parameters is the three-dimensional target position vector. As a consequence, several LOS measurements are required for the localization process. To that end, two options are available: (1) using a single moving passive sensor located on the vehicle [5,6] and (2) an array of sensors pre-placed in a fixed and known position on the seabed [7] or on the ground [8].

Considering the single moving passive sensor option, commonly the target movement is modeled by a constant velocity model, which is then used as a system model in a Kalman framework or any other nonlinear filter [9,10].

One popular method to solve a set of LOS measurements from multiple sensors in a single epoch is the iterative least squares (ILS) algorithm [11]. In general, for a linear scenario, a variety of least squares algorithms may be employed, such as total least squares [12] and generalized least squares [13]. For a nonlinear problem, linearization is applied to convert the nonlinear setup into an iterative algorithm to process the measurements. In the ILS algorithm, given initial conditions, iterations are repeated until a predefined error threshold is reached [10]. ILS can be used for several target tracking related scenarios, like a single moving target tracking with synchronous [8] or asynchronous [14] LOS measurements collected from several passive sensors or multi-target tracking [15]. There, several moving robots along with robots observations, target positions, and velocities are modeled as a graph, which is later modeled and solved as a nonlinear least squares problem. In addition to target tracking, ILS is used also in 3D model compression [16]. Using ILS gives more compact representation while maintaining objects’ geometries and topologies. ILS is also used in image denoising [17] where the noisy image passes through a wavelet filter and then least squares regularization is applied to make the smoothed image similar to the noisy image.

Focusing on the underwater environments, one of the most challenging localization problems concerns the autonomous underwater vehicle (AUV) docking scenario. AUV docking onto a predefined location can be used to transfer data or charge the AUV without pulling it out of the sea. While several works suggest localization using vision-based algorithms, the localization solution assumes the docking panel’s shape is known [18], and in several approaches, lamps are located at the entrance of the docking station to provide clearer images [19]. The performance of vision-based approaches depends on water turbidity and visibility. In other solutions, multiple sensors, both active and passive, are combined, thereby returning more accurate position estimation [20,21]. A combination of vision and non-vision based solutions exists, as well [22].

This paper presents a deep-learning based framework for the bearing-only target tracking process, instead of ILS, named BOTNet: bearing-only tracking network. BOTNet is applicable for any bearings-only target tracking task, regardless of the operating target environment. As a data-driven method, the proposed deep-learning framework offers several advantages over the traditional iterative least squares. To begin with, it is a data-driven approach that implicitly learns the data distribution, then generalizes and predicts the unknown target position based on LOS measurements. In addition, the framework can be focused to learn limited or specific scenario parameters to improve its performance, a feature not available in traditional ILS. Moreover, no initialization process or initial guess is required in contrast to the traditional ILS algorithm. Yet, the proposed network operates with a defined specific input dimension. This translates into a fixed number of LOS measurements. Thus, a different network is required to match each number of expected LOS measurements, unlike the traditional ILS which can operate without any limitations on the number of LOS measurements.

To demonstrate the proposed approach, a scenario of tracking an AUV approaching an underwater docking station is considered. There, several passive sensors (e.g., cameras) are placed near a docking station to enable accurate localization of an approaching autonomous underwater vehicle.

The rest of the paper is organized as follows: Section 2 presents the problem formulation of target location estimation using LOS measurements, Section 3 represents the deep learning based approaches, and Section 4 compares the ILS method and the deep learning based approach, for several test cases. Section 5 gives the conclusions.

## 2. Problem Formulation

To formulate the ILS problem, a scenario of *n* passive sensors measuring LOS measurements to a target with unknown position is considered. Two options for the sensor positions may be considered: (1) all sensor position vectors in some global Cartesian coordinates frame are known, and (2) all sensor position vectors in some local Cartesian coordinates frame are known, for example, relative to one of the sensor positions. In the former, the target position will be determined in the global frame, while, in the latter, in the local coordinate frame. Regardless of the coordinate frame choice, the mathematical formulation is identical. Therefore, it is assumed that all sensor position vectors are known.

Let *n* be the number of sensors, each located in a fixed position in Cartesian coordinates as

(1) $$p_{i}={\left[\begin{array}{ccc} x_{i} & y_{i} & z_{i} \end{array}\right]}^{T}\;\in R^{3},\;i=1,2,\ldots ,n,$$

where $p_{i}$ is the position vector of sensor *i*.

Each sensor provides one LOS measurement of the (unknown) target position, $p_{t}$, represented in Cartesian coordinates as

(2) $$p_{t}={\left[\begin{array}{ccc} x_{t} & y_{t} & z_{t} \end{array}\right]}^{T}\;\in R^{3}.$$

The LOS measurement from sensor *i* is a function of the sensor location and the unknown target location:

(3) $$m_{i}=g\left(p_{t},p_{i}\right)+v_{i},$$

where $v_{i}$ is a random noise modeled as a zero mean white Gaussian noise. The nonlinear function in (3) is defined as

(4) $$g\left(p_{t},p_{i}\right)=\left[\begin{array}{c} \alpha_{i}\\ \epsilon_{i} \end{array}\right]=\left[\begin{array}{c} \arctan\left(\frac{y_{t}-y_{i}}{x_{t}-x_{i}}\right)\\ \arctan\left(\frac{z_{t}-z_{i}}{\sqrt{{\left(x_{t}-x_{i}\right)}^{2}+{\left(y_{t}-y_{i}\right)}^{2}}}\right) \end{array}\right],$$

where $\alpha_{i}$ is the azimuth angle, and $\epsilon_{i}$ is the elevation angle of sensor *i*. The formation of the composite measurement (holding all sensor LOS measurements) is made using a maximum likelihood (ML) criterion by maximizing the likelihood function of the target state at the fusion time $t_{f}$,

(5) $$x\left(t_{f}\right)={\left[\begin{array}{ccc} x(t_{f}) & y(t_{f}) & z(t_{f}) \end{array}\right]}^{T},$$

based on the set of measurements M, which is the probability density function of the measurement set conditioned on $x(t_{f})$; namely

(6) $$\Lambda \left[x\left(t_{f}\right);M\right]≜p\left[M|x\left(t_{f}\right)\right],$$

where Λ(x) is the likelihood function of the unknown parameter vector to be estimated

(7) $${\hat{x}}_{ML}=\underset{x(t_{f})}{\mathrm{argmax}}\Lambda \left[x\left(t_{f}\right);M\right]=\underset{x(t_{f})}{\mathrm{argmax}}\lambda \left[x\left(t_{f}\right);M\right],$$

and

(8) $$\begin{array}{c} \begin{array}{cc} \lambda (x) & =-\ln\Lambda (x)\\ \; & =\sum_{k}{\left[m_{i}-g\left(p_{t},p_{i}\right)\right]}^{T}\times R^{-1}\left[m_{i}-g\left(p_{t},p_{i}\right)\right] \end{array} \end{array}$$

is the negative log-likelihood function [23]. R is the measurement-noise covariance matrix:

(9) $$R=\left[\begin{array}{cccc} \sigma^{2} & 0 & 0 & 0\\ 0 & \sigma^{2} & 0 & 0\\ 0 & 0 & \ddots & 0\\ 0 & 0 & 0 & \sigma^{2} \end{array}\right],$$

where it is assumed all measurements have equal variance. Thus, the ML problem becomes a nonlinear least squares problem, and, consequently, the ML estimate can be obtained using the iterated least squares (ILS) algorithm [10]. To that end, let M be a vector consisting of all measurements arriving from *n* sensors at the same epoch:

(10) $$M=\left[m_{1}^{T},m_{2}^{T},\cdot \cdot \cdot ,m_{n}^{T}\right]\in R^{2n}.$$

The composite measurement (10) is a function of the unknown target position (5). The ILS algorithm, which maximizes (7), yields after the jth iteration the estimate of the target state using [10]

(11) $$\begin{array}{c} {\hat{x}}_{ILS}^{j+1}={\hat{x}}_{ILS}^{j}+{\left[{\left(H^{j}\right)}^{T}R^{-1}H^{j}\right]}^{-1}{\left(H^{j}\right)}^{T}R^{-1}\left[M-g\left({\hat{x}}_{ILS}^{j},p_{i}\right)\right], \end{array}$$

where R is defined in (9). The Jacobian matrix is:

(12) $$H^{j}={\left[{H_{1}}^{T}{H_{2}}^{T}\cdot \cdot \cdot {H_{n}}^{T}\right]}^{T},$$

where $H_{i}$ corresponding to the measurement *i* is

(13) $$H_{i}=\left[\begin{array}{ccc} \frac{\partial \alpha_{i}}{\partial x_{t}} & \frac{\partial \alpha_{i}}{\partial y_{t}} & \frac{\partial \alpha_{i}}{\partial z_{t}}\\ \frac{\partial \epsilon_{i}}{\partial x_{t}} & \frac{\partial \epsilon_{i}}{\partial y_{t}} & \frac{\partial \epsilon_{i}}{\partial z_{t}} \end{array}\right].$$

The partial derivatives in (13) are

(14) $$\frac{\partial \alpha_{i}}{\partial x_{t}}=-\frac{\Delta y_{i}}{{\left(\Delta x_{i}\right)}^{2}+{\left(\Delta y_{i}\right)}^{2}},$$

(15) $$\frac{\partial \alpha_{i}}{\partial y_{t}}=\frac{\Delta x_{i}}{{\left(\Delta x_{i}\right)}^{2}+{\left(\Delta y_{i}\right)}^{2}},$$

(16) $$\frac{\partial \alpha_{i}}{\partial z_{t}}=0,$$

(17) $$\frac{\partial \epsilon_{i}}{\partial x_{t}}=-\frac{\left(\Delta x_{i}\right)\left(\Delta z_{i}\right)}{\left|\right|p_{t}-p_{i}{\left|\right|}^{2}\sqrt{{\left(\Delta x_{i}\right)}^{2}+{\left(\Delta y_{i}\right)}^{2}}},$$

(18) $$\frac{\partial \epsilon_{i}}{\partial y_{t}}=-\frac{\left(\Delta y_{i}\right)\left(\Delta z_{i}\right)}{\left|\right|p_{t}-p_{i}{\left|\right|}^{2}\sqrt{{\left(\Delta x_{i}\right)}^{2}+{\left(\Delta y_{i}\right)}^{2}}},$$

(19) $$\frac{\partial \epsilon_{i}}{\partial z_{t}}=-\frac{\sqrt{{\left(\Delta x_{i}\right)}^{2}+{\left(\Delta y_{i}\right)}^{2}}}{\left|\right|p_{t}-p_{i}{\left|\right|}^{2}},$$

where ||·|| is the Euclidean norm. Finally,

(20) $$\left[\begin{array}{c} \Delta x_{i}\\ \Delta y_{i}\\ \Delta z_{i} \end{array}\right]=\left[\begin{array}{c} x_{t}-x_{i}\\ y_{t}-y_{i}\\ z_{t}-z_{i} \end{array}\right]=p_{t}-p_{i}.$$

The initial estimate $\hat{x}ILS0$ is required for the ILS method and requires the positions of two sensors’ locations and their compatible LOS measurements [24]:

(21) $$\begin{array}{c} \begin{array}{cc} \; & {\hat{x}}_{ILS}^{0}=\left[\begin{array}{c} x^{0}\\ y^{0}\\ z^{0} \end{array}\right]=\left[\begin{array}{c} \frac{y_{2}-y_{1}+x_{1}\tan\alpha_{1}-x_{2}\tan\alpha_{2}}{\tan\alpha_{1}-\tan\alpha_{2}}\\ \frac{\tan\alpha_{1}\left(y_{2}+\tan\alpha_{2}\left(x_{1}-x_{2}\right)\right)-y_{1}\tan\alpha_{2}}{\tan\alpha_{1}-\tan\alpha_{2}}\\ z_{1}+\tan\epsilon_{1}\left|\frac{\left(y_{1}-y_{2}\right)\cos\alpha_{2}+\left(x_{2}-x_{1}\right)\sin\alpha_{2}}{\sin\left(\alpha_{1}-\alpha_{2}\right)}\right| \end{array}\right] \end{array}, \end{array}$$

where ${\left[x_{1},y_{1},z_{1}\right]}^{T}$ is the sensor #1 location in the local Cartesian coordinates, ${\left[\alpha_{1},\epsilon_{1}\right]}^{T}$ are the measured azimuth and elevation angles from sensor #1, ${\left[x_{2},y_{2},z_{2}\right]}^{T}$ is the sensor #2 location in the local Cartesian coordinates, and ${\left[\alpha_{2},\epsilon_{2}\right]}^{T}$ are the measured azimuth and elevation angles from sensor #2.

## 3. Deep Learning ILS

**Figure 1:**
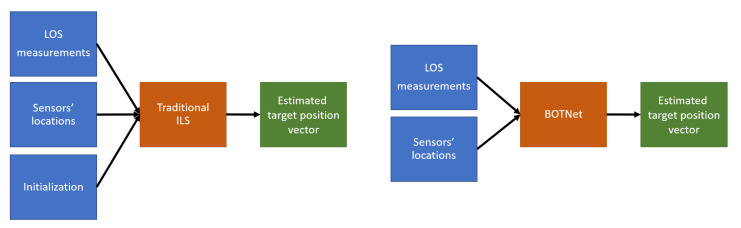
Flowchart comparing the traditional ILS method (**left**) and the proposed approach (**right**).

As described in Section 2, ILS is a common method for parameter estimation based on some model connecting between the measurements and the unknown vector required to be estimated. Linearization is applied to convert the nonlinear problem into an iterative algorithm to process the measurements. To improve the accuracy, the procedure is repeated iteratively with the result from one iteration is used as the linearization point of the next iteration. Hence, the number of iterations influences the accuracy of the solution [11,25]. In the proposed approach, a deep learning framework is set to replace the traditional ILS algorithm. A block diagram showing the traditional ILS and the proposed approach is presented in Figure 1.

The proposed deep learning framework has several benefits:

- The data-driven solution implicitly learns the train data probability distribution to generalize and predict the values of the required unknown parameters based on the given measurements.
- Can be focused to learn specific scenario parameters to improve its accuracy.
- Does not require initialization to start its operation.
- Deep networks have an inherent property of denoising the data [26] from the noisy measurements, which could be utilized to improve performance.

On the other hand, a deep learning architecture operates with a defined specific input dimension. Here, this translates into a fixed number of LOS measurements. Thus, a different network is required for each number of expected LOS measurements. A typical general scenario relevant for the proposed approach consists of:

- An array of passive sensors, each located in a fixed position.
- A moving target.

In this paper, an AUV docking scenario is considered; thus, the passive sensors are located near a docking station, and the moving target is the AUV. Full details of the scenario are provided in Section 4.1.

When constructing and training a deep neural network, the goal is to find an approximation to a function connecting the input to the network (LOS measurements) to its output (target position vector). The approximation is in the form of a deep neural network with specific structure and parameters. Full details of our specific approximation, i.e., BOTNet are described later on in this section.

BOTNet is constructed such that its output is the estimated target position vector ${\hat{p}}_{t}$. The input consists of the sensor locations (in local Cartesian coordinates) and their corresponding LOS measurements. To that end, the measured azimuth and elevation angles are used to construct the LOS vector from sensor *i*:

(22) $${LOS}_{i}=\left[\begin{array}{c} \sin\epsilon_{i}\cos\alpha_{i}\\ \sin\epsilon_{i}\sin\alpha_{i}\\ \cos\epsilon_{i} \end{array}\right].$$

BOTNet structure comprises of fully connected layers. Fully connected networks, also commonly known as deep feedforward networks or feedforward neural networks, are the most basic form of deep neural networks. They assume no relation between the input entries; therefore, they can fit any type of problem.

**Figure 2:**
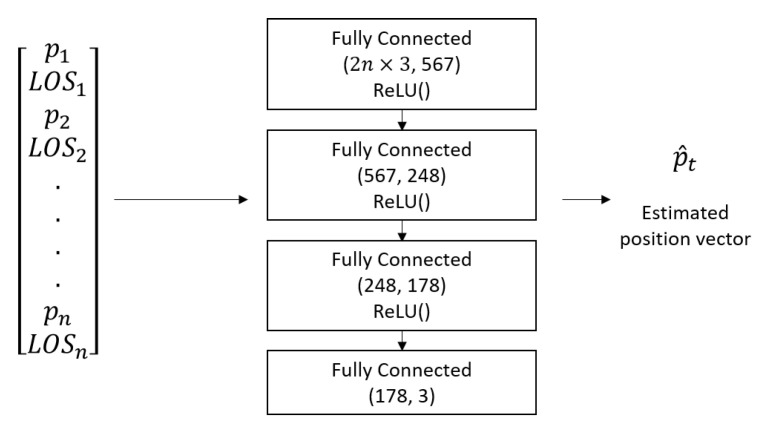
Overview of BOTNet’s input, output, and architecture. Given *n* sensors, network input is 2n vectors, and each one is three-dimensional. For each sensor, the input is both the sensor location and the LOS measurement it produces. Input is converted to BOTNet as a single line of length 6n.

The fully connected layers are arranged in a chain-like structure such that each layer’s input is the output of the previous layer. In such a layer, each node, also named neuron, gets as input the output from all the neurons in the previous layer. The first layer is defined by [27]:

(23) $$h^{\left(1\right)}=g^{\left(1\right)}\left(W^{{\left(1\right)}^{T}}x+b^{\left(1\right)}\right),$$

where x is the input sample, W is the weight matrix, b is the bias vector, and *g* is the activation function. The weights and biases create a mapping between the neurons in the current layer and the neurons from the previous layer. Their exact values are computed during the network optimization process such that the loss function is minimized. The *i*-th layer is defined by:

(24) $$h^{\left(i\right)}=g^{\left(i\right)}\left(W^{{\left(1\right)}^{T}}h^{\left(i-1\right)}+b^{\left(i\right)}\right),$$

where $h^{\left(i-1\right)}$ is the output of the previous layer. Each layer uses the Rectified linear unit (ReLU) as a nonlinear activation function. ReLU is defined as [28]:

(25) g(z)=max{0,z},

where *z* is the input to the activation function. The detailed architecture is described in Figure 2.

The number of layers in a specific feedforward neural network can vary, as well as the number of neurons in each layer. These values, among others, are named hyper-parameter values. The specific values for BOTNet are described in Figure 2 and, later on, in this section. The desired network approximation function should be the closest possible to the real function relating the input and output to the network. This is done by minimizing a loss function associated with the problem at hand. The root mean square error (RMSE) was chosen for the loss function since a regression problem is addressed. The input to the loss function is the estimated target location for sample *i*, ${{\hat{p}}_{t}}^{\left(i\right)}$ and the actual target location for data sample *i*, ${p_{t}}^{\left(i\right)}$, each is a vector of dimension d=3. The loss function is computed on each data sample using:

(26) $$L\left({\hat{P}}_{t},P_{t}\right)=\underset{{\hat{P}}_{t}}{\mathrm{argmin}}RMSE\left({\hat{P}}_{t},P_{t}\right)=\underset{{\hat{P}}_{t}}{\mathrm{argmin}}\sqrt{\frac{1}{n}\sum_{i=1}^{n}\left[\frac{1}{d}\sum_{j=1}^{d}{\left({\hat{p_{t}}}_{j}^{\left(i\right)}-{p_{t}}_{j}^{\left(i\right)}\right)}^{2}\right]},$$

where ${\hat{P}}_{t}$ is the set of n estimated target locations, and $P_{t}$ is the set of the corresponding n actual target locations.

During the training stage of BOTNet, the actual target position $p_{t}$ is given to the network, and BOTNet is optimized to find a set of parameters W,b, such that the loss function is minimized. Since our input dimensionality is considered small, this network’s training process can be fast enough. Hyper-parameter values were optimized using the Bayesian optimization tool [29]. Training consisted of 1000 epochs with a learning rate of 0.0001 and a batch size of 100. During testing, BOTNet has to return an estimate of the target position vector for each sample. This output is compared with the actual target position $p_{t}$, and then metrics, such as RMSE, are computed to examine the network’s ability to estimate the target location for unseen data samples. During this work, several other architectures were examined, but only the best one (in terms of minimum error) is described herein. This is because the goal of this paper is to derive the BOTNet framework, rather than finding the optimal network. This task is left for future work.

## 4. Analysis and Results

Several scenarios are defined to evaluate the performance of the proposed framework with BOTNet and to compare its performance relative to the traditional ILS. Those scenarios are

- The influence of different types of input, which is the representation of the measurements and sensor locations, to the network, is addressed in Section 4.2.
- In Section 4.3, the noise level impact on the localization process is addressed.
- Different test datasets, created within the grid boundaries are used to evaluate the network performance are presented in Section 4.4.
- BOTNet’s ability to generalize unseen data from a test dataset with different distribution, created outside the grid boundaries, is examined in Section 4.5.
- The influence of varying number of sensors, each providing a single LOS measurement, is addressed in Section 4.6.

The performance measure used to evaluate ILS and BOTNet in all of the above scenarios is RMSE (26).

### 4.1. Scenario and Dataset

**Figure 3:**
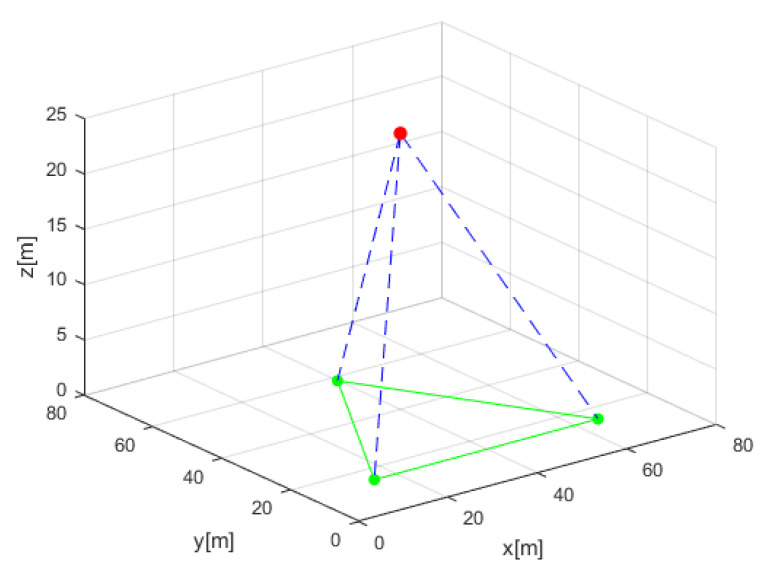
General scenario with a three-sensor configuration (green dots) and AUV (red). Each dashed blue line represents a single LOS measurement. The local coordinate frame is located on the seabed.

A numerical simulation was used to generate the dataset and to perform the comparison to traditional ILS. This resulted in 2800 different AUV locations in the grid. To that end, the following parameters and assumptions are made:

- Number of sensors, denoted by *n*, is equal to 3, each providing a single LOS measurement.
- Sensor locations are fixed on an horizontal plane, forming an equilateral shape with 50 m edge length, as shown in Figure 3.
- The target position varies in the boundaries of [x,y]∈[0,76] meters with step size of four meters in the x or y axes. In the z axis, values of z ∈[10,40] meters with step size of five meters were used.
- All LOS measurements arrive at the same time.
- The true LOS were calculated using plane geometry. To construct the measured LOS, a zero mean white Gaussian noise with a standard deviation of 0.01 rad was added.

The dataset was created as follows:

(1) The AUV location was picked from one of the possible locations described in Section 4.1.
(2) The LOS measurements were generated according to the chosen AUV location and sensor locations (fixed position).

For each given AUV location, the true LOS vector was calculated followed by ten different realizations of the corresponding LOS measurement, resulting in a total of 28,000 LOS measurements in the defined grid. An example of three sensor locations (green) and an AUV location (red) can be seen in Figure 3.

The dataset was split to train and test sets according to the following proportions: 80% and 20% of samples in each set, respectively. Unless stated otherwise, splitting into training and testing sets is performed randomly.

### 4.2. Input Data Normalization

For the localization task at hand, an accurate representation of the data is crucial to prevent localization errors. The basic option to define the input is using the measured azimuth and elevation angles from each sensor. In that manner, the range of the angles could be [0,2π] or [−π,π]. Instead, in this paper the LOS vector measurement (22) is used. This ensures that all values will be ∈[−1,1].

As the input to the network also includes the known sensor positions, two options were examined. In the first one, the sensor locations were not normalized, while, in the second option, they were normalized to the range of [−1,1].

BOTNet with both input types (not normalized and normalized sensor positions) and the ILS algorithm were evaluated on the test dataset. RMSE results are presented in Table 1. As can be seen, both approaches improved ILS performance; in particular, when using normalized sensor locations, an improvement of approximately 40% was achieved. This result shows that, for the given scenario parameters, the proposed BOTNet outperformed the traditional ILS approach.

### 4.3. Noise Sensitivity

**Figure 4:**
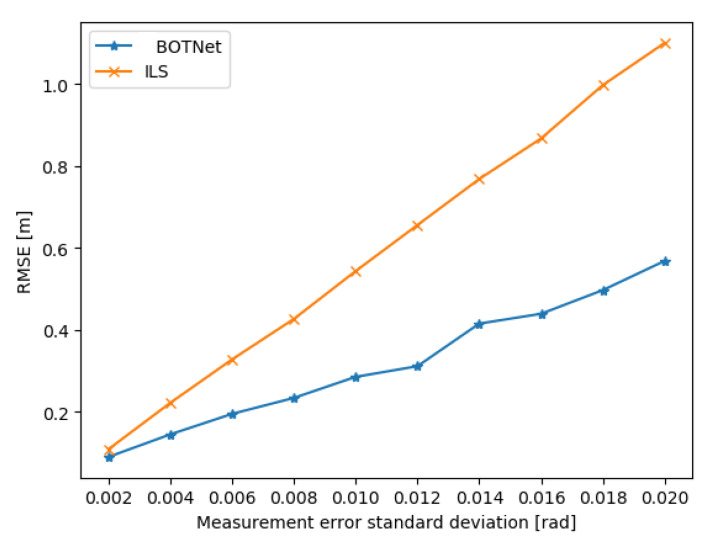
Position error comparison of ILS and BOTNet per standard deviation value.

To check the effect of the noise magnitude σ on the target position error of both ILS and BOTNet, several datasets were created in the same fashion as described in Section 4.1, only with different noise levels. The STD values are in the range of σ∈[0.002,0.02] radians with a step of 0.002 radians between successive values. In total, 10 datasets were created and evaluated. As can be seen in Figure 4, BOTNet outperformed the ILS method for all values of σ. For example, for σ=0.002, a minimum difference of 17.4% was achieved, while, for σ=0.02, the maximum difference was 48.4%.

### 4.4. Dataset Influence

**Figure 5:**
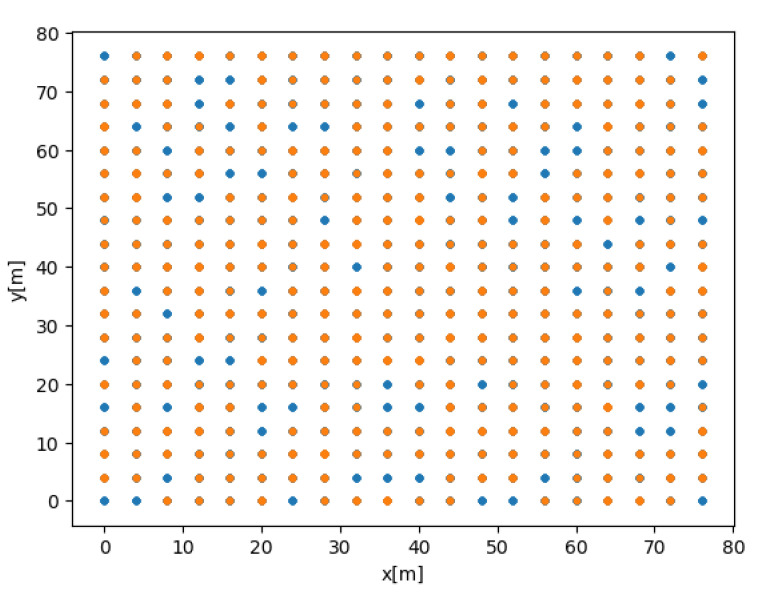
Case B visualization showing possible AUV positions for the train (red) and test (blue) datasets.

Several options for splitting the dataset within the original grid and its influence on BOTNet’s performance are examined. The motivation behind this analysis is to check BOTNet’s ability to estimate the target’s position vector given unseen data, which might have different properties in comparison to samples given to the network during training. To that end, the following cases are considered for the test dataset:

1. Case A : Random split using the grid of possible AUV locations described in Section 4.1.
2. Case B: Random split as in Case A, limited in that all measurements taken from a certain location must appear in the training or testing sets. That is, the test dataset includes AUV locations not present in the training dataset. Case B is visualized in Figure 5, showing possible AUV positions for the train and test datasets in a two-dimensional grid.
3. Case C: Grid offset. This dataset is created using the same guidelines described in Section 4.1 but adding an offset of two meters in the x and y axes for each possible AUV location in the grid.
4. Case D: Random grid. In all previous cases, the grid was chosen using some conditions in a deterministic manner. Here, the possible AUV location is chosen randomly within the grid boundaries as defined in Section 4.1. To that end, in each cell of the original grid, an AUV location is randomly selected.

**Figure 6:**
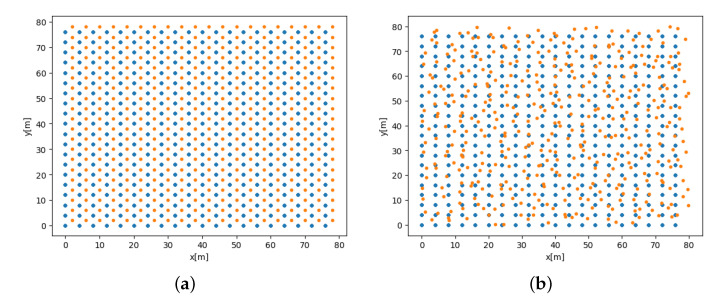
Visualization of the different dataset scenarios: (**a**) refers to case C, and (**b**) refers to case D. Figure shows possible AUV positions for the train (red) and test (blue) datasets.

Cases C and D are visualized in Figure 6.

**Figure 7:**
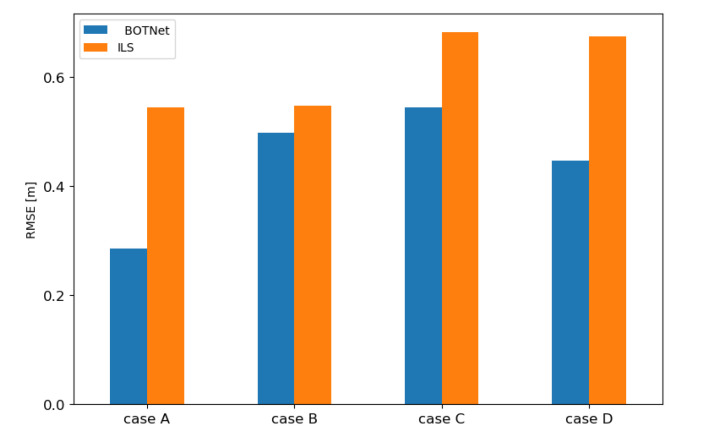
Position RMSE comparison between ILS and BOTNet for the four examined cases.

In each of the described scenarios, BOTNet outperformed ILS, as shown in Figure 7. The largest difference between BOTNet and ILS is 47%, achieved in Case A, and the smallest of 9% was in Case B. For Cases C and D, the difference is 20% and 32%, respectively. This shows that, unlike ILS, BOTNet can generalize and return better AUV location estimations as long as they are within the grid present in the training dataset.

### 4.5. Network Robustness

In this case, the AUV possible locations in the test dataset were created outside the boundaries of the original grid (described in Section 4.1). To that end, the AUV locations were selected by moving it outward from the grid in the following manner: a single face of the three-dimensional rectangular cuboid grid is chosen. From this face, the AUV position was moved using a constant baseline. This procedure was repeated for six faces. For the *x* and *y* axes, the baseline was 40 m, while, for the *z* axis, the baseline was 25 m.

All six possibilities were examined, with three axes moving in the positive or negative directions. This procedure moved the possible AUV locations to a new grid as follows: *x* and *y* axes [80,156] meters in the positive direction and [−80,−4] meters in the negative one; for *z* axis, [60,85] meters in the positive direction, and [−40,−15] meters in the negative one. There, the grid step size is four meters for the *x* and *y* axes, and five for the *z* axis.

For each possible AUV location in this grid, 100 measurements of LOS were simulated to create the test dataset.

**Figure 8:**
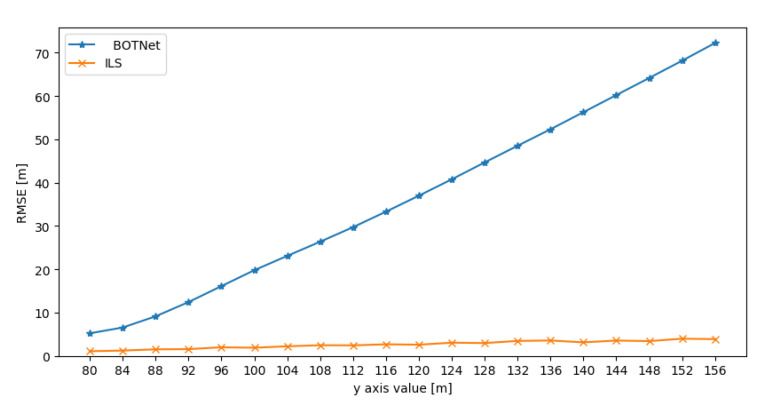
RMSE results for BOTNet and ILS when the AUV set of possible locations moves outward from the original grid in the positive y direction.

**Figure 9:**
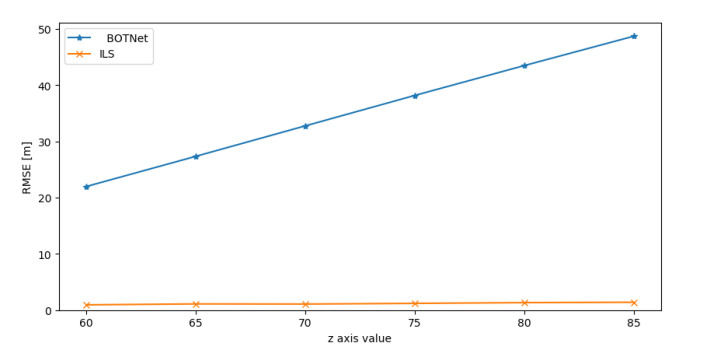
RMSE results for BOTNet and ILS when the AUV set of possible locations moves outward from the original grid in the positive z direction.

Results for the positive *y* (*x* axis is identical to *y* axis) and *z* axes are shown in Figure 8 and Figure 9. The results when the AUV possible locations are in the negative directions of *x*, *y*, and *z* are not presented as the behavior is identical to those of the positive directions.

In all six possibilities, the ILS approach outperformed BOTNet while maintaining the same level of accuracy as achieved when the AUV possible locations were within the grid. On the other hand, BOTNet performance degraded as the AUV position moved outward from the orignal grid.

This result is not surprising since deep neural networks usually return large errors when training and test sets represent data from different distributions, as in this case. Thus, to overcome such situations, the training grid should be extended to include all possible AUV locations.

### 4.6. Influence of Number of Sensors

**Figure 10:**
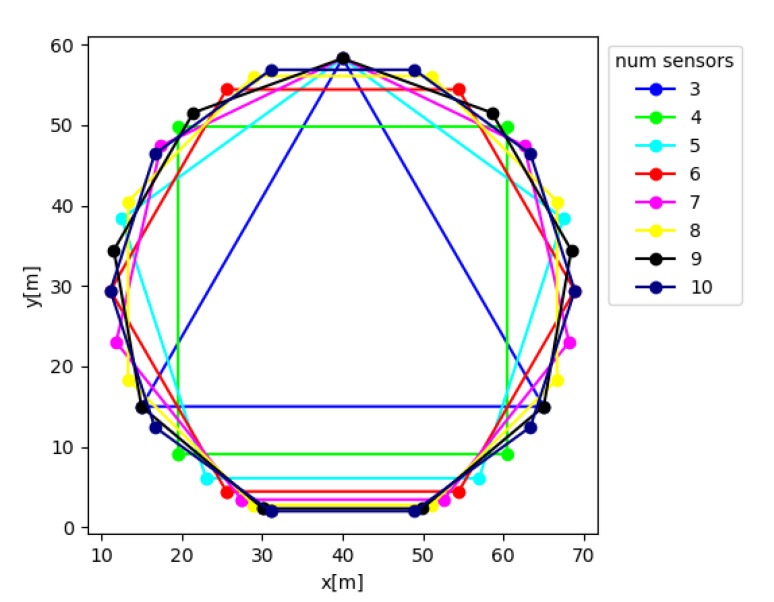
Sensor locations per number of sensors. Sensor locations are at the nodes of each polygon in the horizontal plane.

BOTNet is evaluated here against different datasets, each of which is created for a given number of sensors N∈[3,10], with each contributing a single LOS measurement. For each possible number of sensors *N*, sensor locations were set to be the nodes of each polygon, in the horizontal plane, with *N* edges, as illustrated in Figure 10.

**Figure 11:**
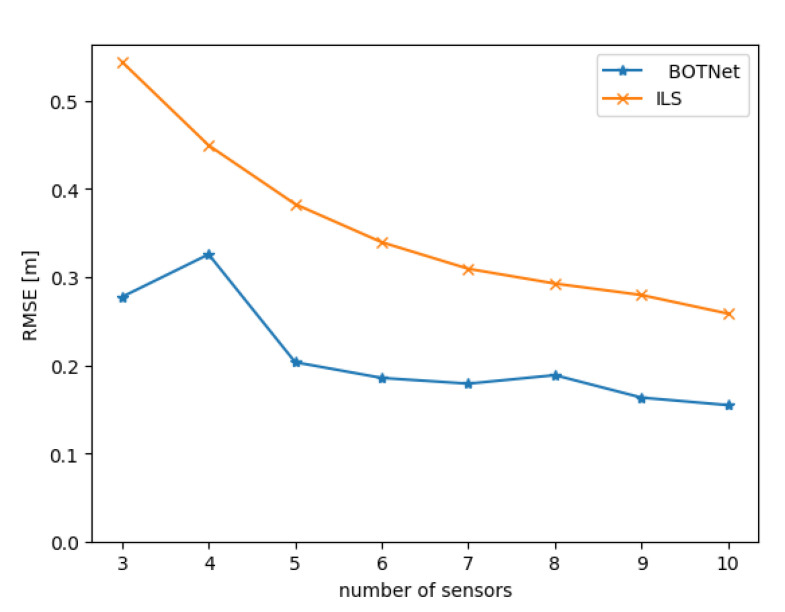
Position RMSE for both BOTNet and ILS as a function of the number of sensors, where each sensor provides a single LOS to the AUV.

Figure 11 presents the position RMSE for both ILS and BOTNet as a function of the number of sensors. For both approaches, as the number of sensors increases (more LOS measurements), the performance increased. Regardless of the number of sensors, BOTNet obtained better performance than ILS.

## 5. Conclusions

In this paper, an alternative method for estimating an unknown target location from noisy measurements arriving from a multisensor passive system was suggested instead of the ILS approach.

The proposed framework, BOTNet, uses a deep neural network created for the localization task. The network receives the known sensor locations and their LOS measurements and outputs the estimated target position vector.

Using the proposed framework has several advantages over the ILS approach. First, it is based on a deep learning algorithm, which is a data-driven approach. Therefore, it implicitly learns the data’s distribution and can learn specific parameters relevant to a certain scenario. In addition, deep networks enjoy an inherent property of denoising the noisy measured LOS, which improves the accuracy of the proposed approach. Finally, in contrast with ILS, no initial guess or initialization process is required. On the other hand, a data-driven approach requires a preliminary stage of collecting or constructing the dataset and then training the network. In addition, the input to the network or ILS is the sensors’ known locations and their LOS measurements. While, for the ILS, the number of sensors can vary during operation, it must remain fixed for BOTNet as the network is trained for a specific input dimension.

To demonstrate the benefits of the proposed approach, an AUV docking scenario was considered. There, passive sensors are placed near the docking station to provide LOS measurements to the approaching AUV.

Several cases were examined to evaluate BOTNet. To that end, a three-dimensional rectangular cuboid grid representing possible AUV locations near the docking station was created. In all examined cases, BOTNet achieved a lower RMSE error compared with ILS, except for the scenario when the AUV locations were taken out of the grid. The latter can be circumvented by defining a larger grid for the training dataset. Although the paper focuses on an AUV localization task, BOTNet can be easily adapted for other bearing-only tracking scenarios, such as tracking air platforms or tracking a member in a formation, or other domains that currently use an iterative least squares based approach, like 3D model compression.

The implementation of the proposed framework is available at https://github.com/Hadar-Sha/BOTNet, accessed on 31 May 2021.

## Figures and Tables

**Table 1:** RMSE of the test dataset for ILS and BOTNet approaches.

Approach	RMSE [m]
BOTNet with normalized sensor positions	0.285
BOTNet with unnormalized sensor positions	0.496
ILS	0.55

## Data Availability

The implementation of the proposed framework is available at https://github.com/Hadar-Sha/BOTNet.
